# Oral Tolerance Induction in Experimental Autoimmune Encephalomyelitis with *Candida utilis* Expressing the Immunogenic MOG35-55 Peptide

**DOI:** 10.1371/journal.pone.0155082

**Published:** 2016-05-09

**Authors:** Christoph Buerth, Anne K. Mausberg, Maximilian K. Heininger, Hans-Peter Hartung, Bernd C. Kieseier, Joachim F. Ernst

**Affiliations:** 1 Institute of Molecular Mycology, Department Biology, Heinrich-Heine-University, Düsseldorf, Germany; 2 Research Group for Clinical and Experimental Neuroimmunology, Department of Neurology, Medical Faculty, Heinrich-Heine-University, Düsseldorf, Germany; Wayne State University, UNITED STATES

## Abstract

Multiple sclerosis (MS) is an autoimmune disease that attacks myelinated axons in the central nervous system. Induction of oral tolerance is a potent mechanism to prevent autoimmunity. The food yeast *Candida utilis* was used to test the therapeutic potential of oral tolerance induction in an animal model of human multiple sclerosis (MS). We constructed a *C*. *utilis* strain, which displays a fusion peptide composed of the encephalitogenic MOG_35-55_ peptide and the *C*. *utilis* Gas1 cell wall protein on its surface.By immunizing mice with MOG_35-55_ peptide experimental autoimmune encephalomyelitis (EAE) was induced in a mouse model. Feeding of mice with *C*. *utilis* that expresses MOG_35-55_ peptide on its surface was started seven days prior to immunization and was continued for ten days. Control animals were treated with wild-type fungus or left untreated. Untreated mice developed first clinical symptoms ten days post immunization (p. i.) with an ascending paralysis reaching maximal clinical disability at day 18 to 20 p. i.. Treatment with the wild-type strain demonstrated comparable clinical symptoms. In contrast, oral gavage of MOG_35-55_-presenting fungus ameliorated the development of EAE. In addition, incidence as well as maximal clinical disease severity were significantly reduced. Interestingly, reduction of disease severity also occurred in animals treated with heat-inactivated *C*. *utilis* cells indicating that tolerance induction was independent of fungal viability. Better disease outcome correlated with reduced demyelination and cellular inflammation in the spinal cord, lower T cell proliferation against rechallenge with MOG_35-55_ and more regulatory T cells in the lymph nodes. Our data demonstrate successful that using the food approved fungus *C*. *utilis* presenting the immunogenic MOG_35-55_ peptide on its surface induced an oral tolerance against this epitope in EAE. Further studies will reveal the nature and extent of an anti-inflammatory environment established by the treatment that prevents the development of an autoimmune disorder affecting the CNS.

## Introduction

Multiple sclerosis (MS) is an autoimmune disease affecting the central nervous system (CNS) and one of the commonest causes of neurological disability in young adults [1]. As a model system for MS, experimental autoimmune encephalomyelitis (EAE) is used since it shares some histopathological as well as immunological features of this human disease [2]. EAE can be induced by immunization with myelin components and myelin protein peptides, e.g. myelin oligodendrocyte glycoprotein (MOG)_35-55_. Autoaggressive immune cells infiltrate the CNS resulting in demyelination followed by remyelination or axonal loss [3].

Oral (mucosal) tolerance is a special form of peripheral tolerance suppressing cellular and/or humoral immune responses induced by oral administered antigens, taking place in the gut-associated lymphoid tissue (GALT) [4]. It also prevents inflammatory responses to the microbiome and may also have evolved to avoid hypersensitivity reactions to food [5]. It may also be used to prevent autoimmunity by feeding target antigens [6].

One of the major problems of feeding an antigen is the source, amount and purity of the given antigen. A possibility to circumvent these problems is the administration of yeasts presenting the antigen on their surfaces, specifically on their cell walls. Using yeasts it is easy, to adjust the amount of antigen and the risk of administering toxins, viruses and prions, co-purified with the antigen, is reduced [7, 8]. In previous studies it was shown that feeding mice or rats with MBP or fragments of it suppressed EAE [9,10]. When microorganisms expressing myelin antigens intracellularly were fed, oral tolerance against the produced antigen was induced in animal models [11,12]. Surface display, in which a protein sequence is fused to an anchor protein and attached to the cell surface of an organism, exhibits major advantages compared to conventional secretion systems. Using such cells, which can be (re-)used as a biocatalysts and promote increased protein stability, it is much more cost-efficient to develop vaccines and antibodies [13–15]. In case of oral tolerance induction and oral vaccination, yeast surface (cell wall) display is a convenient method to administer potential antigens to the host immune system (21, 23).

*C*. *utilis*, also known as *Torula* yeast, is an anamorph of *Cyberlindnera jadinii* [16,17] and has been classified as a GRAS (generally recognized as safe) organism by the Food and Drug administration (FDA). It has been used since the beginning of the 20^th^ century as a fodder yeast and as a food additive. *C*. *utilis* is known to efficiently secrete proteins to the culture media [18] and recently, Kunigo *et al*. [19,20] also showed that heterologous proteins fused to the *C*. *utilis* Gas1 cell wall protein are presented in an active form on the fungal cell surface. The complete genome sequence of *C*. *utilis* has been determined and revealed a triploid genome [17,18,21].

In this study we show that by continuous oral administration of a MOG-presenting *C*. *utilis* strain an oral tolerance against the MOG-antigen is generated that significantly reduces the incidence as well as the maximal clinical score of EAE in mice. In addition, even the administration of heat-inactivated MOG-expressing *C*. *utilis* cells led to oral MOG tolerance. This is the first study showing the potential of antigen-presenting yeast cells for treating auto immune diseases and suggests a new approach for induction of oral tolerance in human MS patients.

## Materials and Methods

### Strains and media

*C*. *utilis* wild-type strain DSMZ2361 (ATCC9950), obtained from Deutsche Sammlung für Mikroorganismen und Zellkulturen (DSMZ; Braunschweig, Germany) was used in this study. *C*. *utilis*, also known as *Torula* yeast has been classified as a (generally recognized as safe) organism by the Food and Drug administration (FDA; http://www.fda.gov/Food/IngredientsPackagingLabeling/GRAS/MicroorganismsMicrobialDerivedIngredients/default.htm). Strain MKCu1 was used as control [19]. Yeast strains were grown in YPD media (1% yeast extract, 2% peptone and 2% glucose) at 30°C on a horizontal shaker (110 rpm). To select *C*. *utilis* transformants media were supplemented with 10 μg/ml Nourseothricin (NST; Jena Bioscience, Jena, Germany). For identification of *C*. *utilis* cells in fecal pellets, the pellets were resuspended in PBS and plated out in a serial dilution on agar plates supplemented with 10 μg/ml NST and 100 μg/ml ampicillin (life technologies, Darmstadt, Germany) to prevent bacterial growth. *Escherichia coli* TOP10' (life technologies) cells were used for plasmid construction and were grown in LB media at 37°C supplemented with 100 μg/ml of ampicillin.

### Plasmid construction

**Fig 1:**
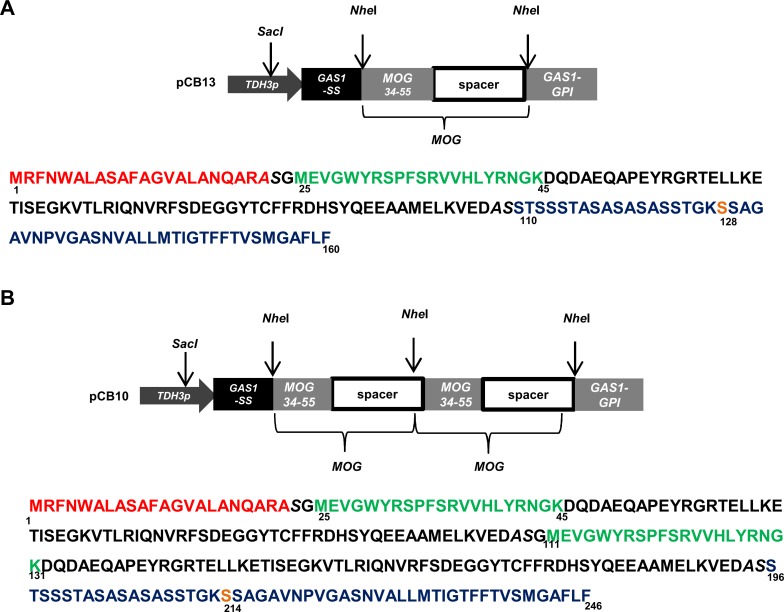
MOG expression vector. **(A)** Scheme of the expression unit of the single MOG plasmid pCB13 and the corresponding encoded amino acid sequence. **(B)** Scheme of the tandem MOG expression plasmid pCB10 and amino acid sequence. The coding sequence for the MOG epitope was inserted between the secretion signal sequence (red) and GPI anchor sequence (blue) of the *C*. *utilis GAS1* sequence. The immunogenic MOG epitope (green) is linked to the *GAS1* GPI sequence with a non immunogenic MOG spacer sequence (black). The potential GPI attachment site (ω) is highlighted in orange. *Nhe*I restriction sites are in *italics*.

The coding sequence for the *Mus musculus* MOG_34-114_ peptide was synthesized based on 29 genes from the Kazusa database (www.kazusa.jp) and were provided in plasmid pMOG from GeneArt (Darmstadt, Germany). The *Mus musculus MOG* coding sequence included the immunogenic MOG_35-55_ epitope and the adjacent 59 amino acid long, non immunogenic spacer sequence. Plasmids pCB13 and pCB10 were constructed by introducing the coding sequence for the MOG_34-114_ peptide between the *Nhe*I restriction sites in plasmid pCB3 [19]. Two plasmids, one encoding for a single MOG peptide (pCB13) (Fig 1A) and a second, encoding for a double MOG peptide (pCB10) (Fig 1B) were constructed. These *MOG* sequences are under the control of the constitutive Cu*TDH3* promoter. The N-terminal secretion signal of Gas1 and the C-terminal Gas1 GPI-sequence were used to secrete the protein and link the peptide to the cell wall, respectively (Fig 1A and 1B). Plasmids were linearized in the *TDH3* promoter region by cutting with *Sac*I to direct genomic integration in *C*. *utilis* and transformants were selected on agar plates containing 10 μg NST/ml. Strains were designated as CBCu7 and CBCu8 carrying plasmid pCB13 or pCB10, respectively.

### Chromosomal plasmid integration and plasmid stability

Chromosomal plasmid integration in strain CBCu8 was confirmed with Southern blot analyses as described earlier [19]. To generate chromosomally stable *C*. *utilis* transformants, strain CBCu8 was repeatedly incubated for 50 generations in YPD-media either with or without selective pressure (10 μg/ml NST). Cells were harvested, washed with PBS, diluted and plated out on YPD agar-plates (2 d, 30°C). 100 colonies were picked and plated out on YPD agar-plates containing 10 μg/ml NST and grown for 2 d at 30°C. Cells were counted and the ratio of NST-resistant to NST-sensitive cells was calculated. One of these NST-resistant cells was used for further experiments (strain CBCu17).

### Immunoblot analysis

Immunoblotting was performed as described earlier (17). Briefly, cell cultures were harvested and washed twice with PBS. An appropriate amount of cells was separated on a SDS-PAGE gel (4–20% acrylamide). Proteins were transferred overnight onto a PVDF membrane (Merck MilliPore, Darmstadt, Germany), afterwards the membrane was blocked (5% non-fat dried milk powder in TBS) and washed with TBST (TBS with 0.2% Tween20). MOG peptides were detected with a primary mouse anti-MOG_35-55_ antibody (1/1000 in TBST) (Aviva Systems Biology, San Diego, US) and a secondary HRP-conjugated anti-mouse antibody (Pierce Biotechnology, Rockford, USA). Protein bands were visualized using the LAS4000 CCD-camera (GE Healthcare, Freiburg, Germany).

### Immunofluorescence

Immunofluorescence was done as described earlier [19]. Briefly, stationary yeast cells were washed and resuspended in S-buffer (50 mM HEPES, 1.2 M sorbitol, pH 7.5). Glass slides were treated for 2 min with 15 μl 0.1% poly-lysine solution and washed with dH_2_O three times. 20 μl of the cell suspension were loaded on the slide and cells were fixed by incubation for 5 min. Unfixed cells were removed by washing four times with PBS. 20 μl of blocking solution (2% skimmed milk powder in PBS) was added and slides were incubated for 15 min. Afterwards, a primary mouse anti-MOG_35-55_ antibody (1/500 dissolved in blocking solution) was added and incubation was continued for at least 90 min. After four washing steps a secondary, FITC-conjugated anti-mouse antibody (1/10,000 in PBS) was added and the slide was incubated for 90 min in dark. The washing step was repeated four times and 20 μl of a 1 μg/ml 4′,6-diamidin-2-phenylindol (DAPI) solution was added to stain chromosomal DNA. All following steps were performed in dark to prevent bleaching. After incubation for 10 min the solutions were removed by washing the slides four times with PBS. "Pro long gold antifade" solution (life technologies) was added according to the manufacturers' protocol to prevent specimen from bleaching. Samples were observed with an Axioskop 40 florescence microscope (Zeiss, Cologne, Germany).

### EAE induction in C57BL/6 mice

All mice were housed under specific pathogen-free conditions in the animal research facility of the University of Duesseldorf according to EU directive 2010/63/EU. Animal experimentation was approved by local state authorities (Landesamt fuer Natur, Umwelt und Verbraucherschutz Nordrhein-Westfalen). Immunization protocols were choosen to prevent that animals became severely sick or died at any time before experimental endpoints. To minimize stress, pain and damage of mice, all animals were handled and treated according to EU directive 2010/63/EU on the protection of animals used for scientific purposes. Animals were daily monitored and termination criteria were definded to protect animals from pain and stress. All animals were sacrificed by cervical dislocation.

To induce active EAE, female C57BL/6 mice (8 weeks, Janvier, Le Genest-Saint-Isle, France) received subcutaneous injections of 200 μg of MOG_35–55_ (Biotrend, Cologne, Germany) in complete Freund's adjuvant (CFA; BD Biosciences, Heidelberg, Germany), supplemented with Mycobacterium tuberculosis H37RA (5 mg/ml) (BD) and 500 ng pertussis toxin (Merck Millipore, Darmstadt, Germany) on d0 and d2. The following EAE score was applied [22]: 0 = no clinical signs; 1 = tail paralysis; 2 = hind limb paresis; 3 = hind limb paralysis; 4 = fore limp paresis; 5 = moribund.

### Feeding of *C*. *utilis*

Strain CBCu17 was incubated in YPD-NST media at 30°C for 72 h, harvested and washed twice in PBS. The pellet was resuspended in PBS and the cell number was adjusted to 1x 10^9^ cells/ml using a haemocytometer (Marienfeld, Lauda-Königshofen, Germany). For heat inactivation of CBCu17, cells were frozen overnight at -80°C and the next day the cell suspension was incubated at 70–80°C for 30 minutes. Inactivation was verified by plating out 100 μl of cell suspension on YPD-NST agar plates. For induction of an oral MOG_35-55_ tolerance daily feeding with 1x 10^8^ cells was started 7 days before EAE induction and was continued for 10 days. Control animals received either the similar amount of wild-type *C*. *utilis* (DSMZ2361) or were left untreated.

### T cell proliferation assay and flow cytometry

Spleens of mice were dissected under sterile conditions and passed through a 40 μm cell strainer followed by ammonium chloride based erythrocyte lysis (BD Biosciences, Germany). Derived splenocytes were cultured in flat bottom 96-well plates in standard T cell medium (IMDM with 5% FCS, 2 mM L-glutamine and 50 μM 2-ME, life technologies). To restimulate cells, MOG_35-55_ was added during the culture period with increasing concentration from 0 to 100 μg/ml. T cell proliferation was measured via [^3^H] thymidine incorporation during the last 24 h of a four day incubation. Counts per minute (cpm) of quadruplicate test cultures ± SEM were determined using liquid scintillation counting (BetaPlate1205, Perkin Elmer, Boston, US). Stimulation index was calculated as ratio of the cpm at the indicated MOG_35-55_ concentrations to the proliferation of cells in the absence of MOG_35-55_. Flow cytometry was performed from homogenized lymphoid organs. Cells were stained for cell surface CD4 (L3T4, APC or Pacific Blue labelled), CD25 (3C7, PerCP labelled) and intracellular FoxP3 (MF23, Pacific Blue labelled) using the FoxP3 staining buffer set (all from BD Biosciences). Flow cytometry was performed using a FACSCanto II flow cytometer (BD Biosciences).

### Histology

At day 35 p.i. animals were sacrificed and perfused first with PBS and 4% PFA. Spinal cords were dissected, post-fixed with paraformaldehyde overnight and paraffin-embedded. Then 7 μm sections (standard microtome HM355S; Microm, Walldorf, Germany) were stained with luxol fast blue (LFB, Sigma Aldrich) and nuclear fast red (Sigma Aldrich) according to manufacturer’s instructions and slices were covered using Roti-HistoKit (Roth). For quantitative analysis of demyelination the area of LFB- stained sections of photographed images (Axioplan 2, Zeiss, Cologne, Germany) was measured by Fiji/ImageJ 1.46j software (NIH, Bethesda, US) and the area of demyelination was calculated as percentage of the white matter area within a given section. For quantitative analysis of infiltration sections DAPI positive cell nuclei were automatically counted within the white matter using the analyze particle tool of the Fiji/ImageJ 1.46j software.

### Data analysis

Flow cytometry data were analyzed using FlowJo software (TreeStar, Ashland, US). Data were statistically analyzed using GraphPadPrism 5.0 (GraphPad Software, La Jolla, US). The Wilcoxon-Mann-Whitney was used to test for statistically significant differences in clinical score values. Student's t-test for unrelated samples was used to test for statistically significant differences in all other analyses. Differences were considered significant at p-values <0.05.

## Results

### MOG production in *C*. *utilis*

Plasmids pCB13 and pCB10, encoding for a fusion containing a single MOG peptide (Fig 1A) and two tandem MOG peptides (Fig 1B), respectively, were linearized in the *TDH3* promoter sequence and chromosomally inserted into *C*. *utilis* DSMZ2361. By Southern blotting the integration of plasmids into the *TDH3* locus was verified (S1A and S1B Fig). Furthermore, transformants stably maintaining the expression plasmid in the absence of NST selection were identified (S1C Fig) and used in animal experiments.

**Fig 2:**
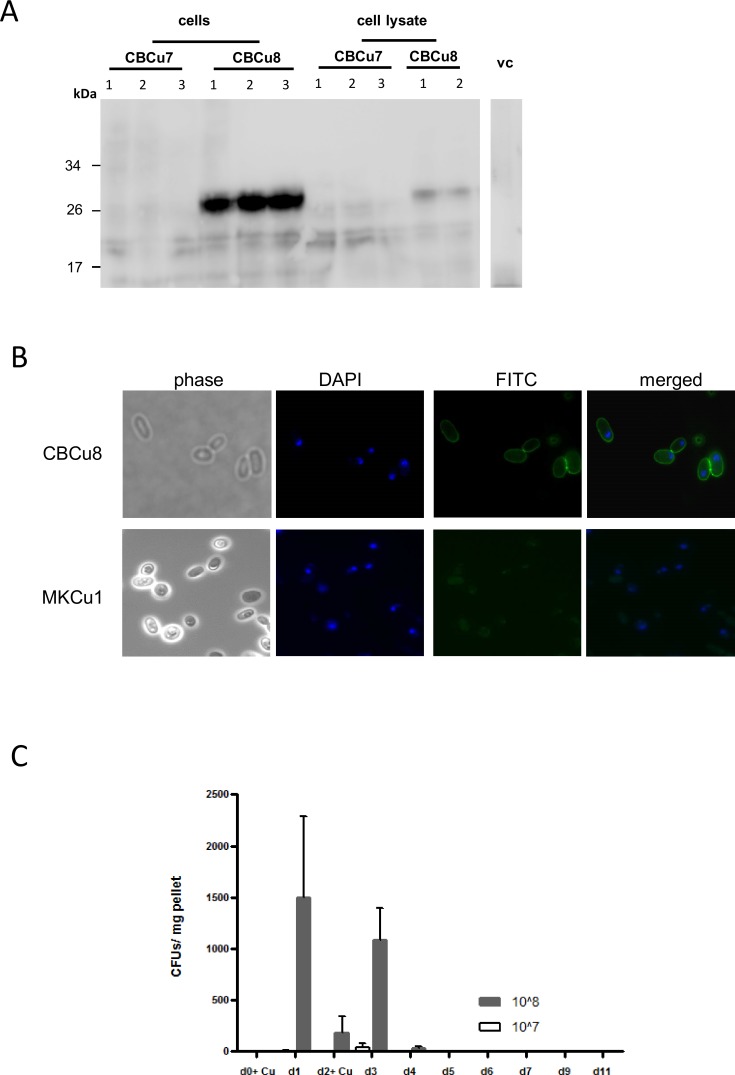
MOG production and localization in *C*. *utilis*. **(A)** Transformants of CBCu7 and CBCu8 were incubated for 72 h in YPD medium supplemented with 10 μg/ml Nourseothricin. Cells were harvested and washed two times with PBS. 20 μl of cells were loaded on a SDS gel (4–20% acrylamide) and the MOG peptide was detected in an immunoblot with a mouse anti-MOG_35-55_ antibody and a secondary horseradish peroxidase coupled anti-mouse antibody. Protein masses are indicated in kDa. Strain MKCu1 (vc) was used as control. Lane 1–3: three independent transformants. **(B)** Strain CBCu8 was incubated for 72 h in YPD medium and MOG was detected on the surface of non permeabilized cells with a mouse anti-MOG_35-55_ antibody and a secondary FITC-coupled anti-mouse antibody. Nuclear DNA was stained with 4′,6-Diamidin-2-phenylindol (DAPI). Strain MKCu1 (*TDH3*p) was used as negative control. **(C)** Feeding protocol. On day 0 (d0) fecal pellets were collected and one group (n = 6) was administered with 1x10^7^ CBCu17 cells, another group with 1x10^8^ cells. Fecal pellets were collected every 24 hours and on day 2 (d2) mice were fed with CBCu17 cells again. On day 11 the experiment was stopped. The collected fecal pellets were plated out on YPD agar plates containing ampicillin (100 μg/ml) and Nourseothricin (10 μg/ml) to prevent bacterial growth and colony forming units were counted. Mean values of three measurements and standard deviations are shown.

Interestingly, in strain CBCu7 (carrying pCB13) no MOG peptides were detected in Western Blot analysis, neither in cytosolic fractions nor in cell debris, whereas in three transformants of CBCu8 (carrying pCB10) a prominent cell-associated MOG signal was detected at a size of about 27 kDa (Fig 2A). The surface localisation of the MOG-Gas1 fusion-protein in CBCu8 was verified with immunofluorescence microscopy in non-permeabilized yeast cells grown in YPD for 72 h. A homogenous distribution of the MOG peptide was detected on the cell surface of CBCu8 (Fig 2B), while in control strain MKCu1 (vector control) no FITC-fluorescence was detected (Fig 2B). A derivative of strain CBCu8 stably maintaining the expression plasmid was identified and designated CBCu17.

### Feeding mice with *C*. *utilis*

The ability of *C*. *utilis* to survive or to grow in the intestinal tract of mice was determined in pre-tests. Two groups (n = 6) of 6–8 weeks old C57BL/6 mice were fed on day 0 and on day 2 with either 1x 10^7^ or 1x10^8^ CBCu17 cells. To promote growth of *C*. *utilis* in the intestinal tract antibiotics (tetracycline 50 μg/ml) were added to drinking water. Fecal pellets were collected every 24 hours and screened for the presence of CBCu17 cells on selective YPD agar plates containing 10 μg/ml NST and 100 μg/ml ampicillin to prevent bacterial growth. In fecal pellets of mice, which were fed with 10^7^ CBCu17 cells, no CBCu17 colony forming units (CFUs) were observed throughout the whole feeding process. In fecal pellets of mice which were given 10^8^ cells about 1,500 CFUs per mg pellet were detected the day post first feeding (Fig 2C). Interestingly, the amount of cells decreased when feeding was intermitted for one day (Fig 2C, d2). The amount of CFUs increased when mice were fed again with CBCu17 cells on the following day, but decreased again when feeding was stopped (Fig 2C). Since antibiotics itself can ameliorate EAE, we omitted the addition of antibiotics to the drinking water, because no influence on growth of *C*. *utilis* in the gut was observed without tetracycline (data not shown). Additionally, we examined histological samples for the presence of CBCu17 cells in the duodenum, ileum, colon and caecum at day five. CBCu17 cells were only detected in the caecum but not in other parts of the intestinal tract (data not shown). These results indicate that a minimal amount of 10^8^ cells is needed to pass the intestinal tract and that *C*. *utilis* is not able to persist in the intestinal tract. Even a continuous feeding of 5 days with 10^8^ *C*. *utilis* cells did not lead to colonization of the fungus in the gut (data not shown). Collectively, these results indicate that a continuous feeding of a certain minimum amount of *C*. *utilis* is needed to be detectable in the gastrointestinal tract.

### Effect of oral administration of MOG_35-55_ expressing *C*. *utilis* on clinical course of EAE

**Fig 3:**
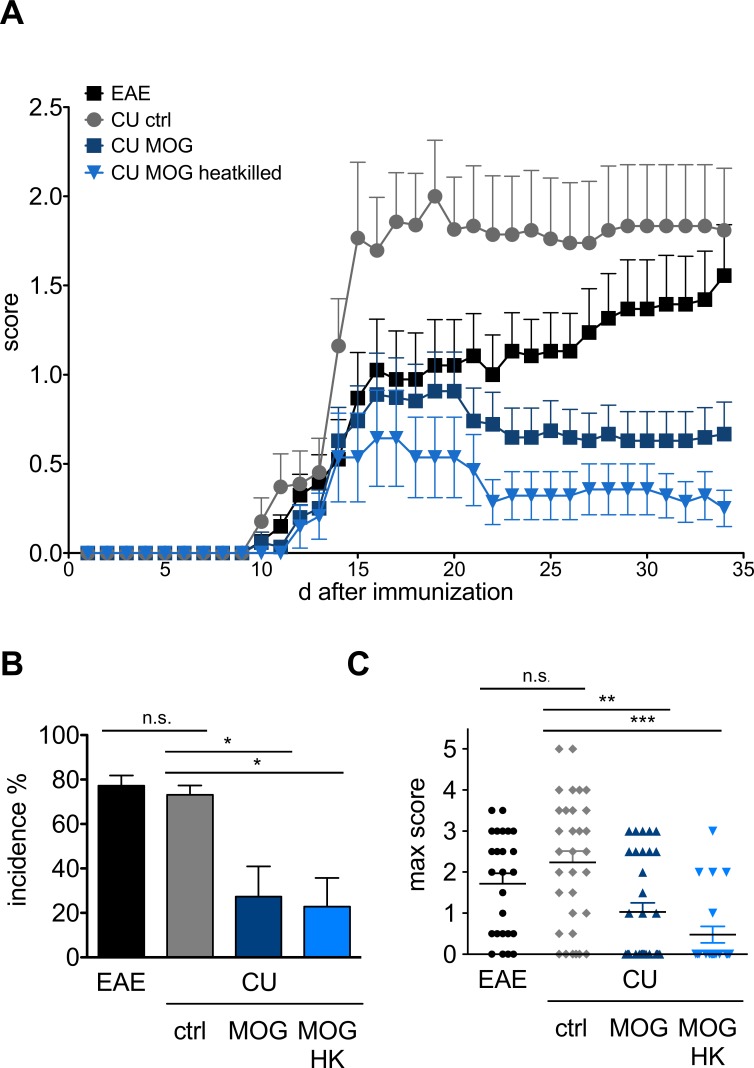
Clinical manifestation of EAE in mice after oral gavage of *C*. *utilis* expressing MOG_35-55_. **(A)** Mean clinical score of four independent experiments testing the preventive potential of *C*. *utilis* expressing MOG_35-55_. C57BL/6 mice (n = 6–8) were immunized and 1.5 x 10^8^ *C*. *utilis* were applied daily by oral gavage for 7 days prior immunization for 10 days. *C*. *utilis* expressing MOG_35-55_ (CBCu17) was tested either alive or heat killed. Control groups received 1.5 x 10^8^ wild-type *Candida utilis* (ctrl.) or no cells (EAE). **(B)** Incidence of EAE and **(C)** maximal score at the peak of the disease after oral gavage of *C*. *utilis* to C57BL/6 mice in the indicated groups. Incidence was calculated as percentage of mice that displayed clinical symptoms of a score of one for more than two days deviated to number of immunized mice in the given groups for each experiment. Clinical scoring was assessed daily using the following system: 0 = no clinical signs; 1 = tail paralysis; 2 = hind limb paresis; 3 = hind limb paralysis; 4 = fore limp paresis; 5 = death. Depicted is the mean ± SEM of four independent experiments. Asterisks indicate significance (* p < 0.05; *** p < 0.01 *** p < 0.001) student’s t test.

The effect of oral administration of MOG_35-55_ expressing *C*. *utilis* on the clinical course of EAE was tested in two control cohorts receiving wild type *C*. *utilis* or left untreated and mice fed with living or heat-killed *C*. *utilis* expressing MOG_35-55_ (Fig 3A). Untreated mice developed first clinical symptoms visible ten days post immunization (p.i.) with an ascending paralysis reaching maximal clinical disability at day 18 to 20 p.i.. Treatment with the control yeast strain not expressing the MOG peptide resulted in comparable clinical symptoms at the endpoint of the experiment. In contrast, oral gavage of MOG_35-55_ expressing *C*. *utilis* resulted in a significant reduction of the clinical signs of EAE. In the two control groups (EAE and wild-type *C*. *utilis*) the incidence was 77.3 ± 4.5 and 73.1 ± 4.3, respectively, with a maximal clinical score of 1.7± 0.2 and 2.2± 0.3 (Fig 3B and 3C). When MOG was present on the surface of *C*. *utilis* the incidence was significantly reduced to 27.3 ± 13.7 and 22.3 ± 12.9 in cohorts receiving living (CU MOG) or heat killed (CU MOG HK) fungi and the maximum score was reduced (1.0 ± 0.2 and 0.5 ± 0.2), respectively (Fig 3B and 3C).

### Myelination and cellular infiltration in the CNS after oral administration of MOG_35-55_ expressing *C*. *utilis*

**Fig 4:**
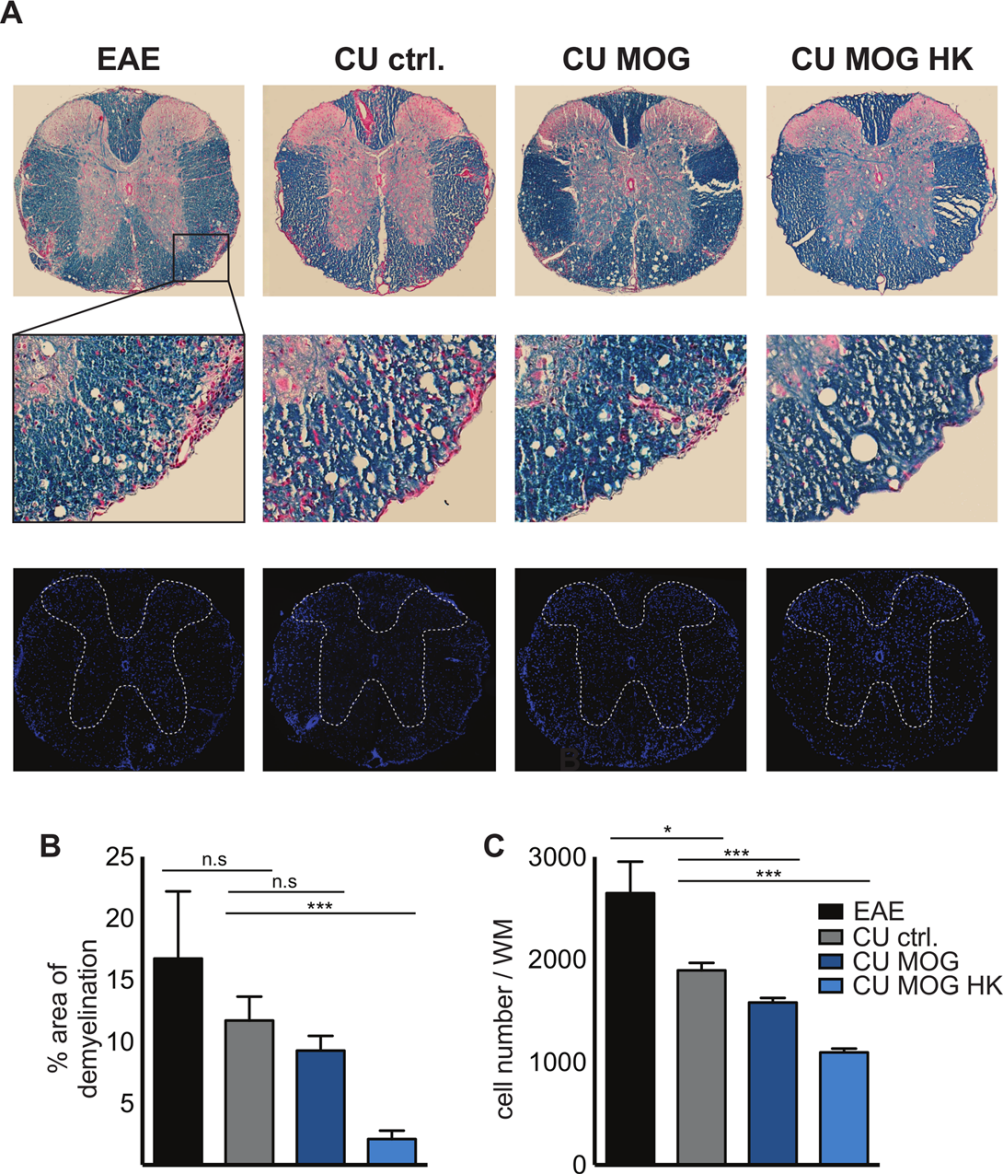
Myelination and cellular infiltration in the CNS of after oral gavage of *C*. *utilis* expressing MOG_35-55_. **(A)** Representative histopathological stainings and quantifications of myelination and cellular infiltration. Spinal cord sections from day 35 p.i. were stained with luxol fast blue (LFB, myelin) and nuclear fast red for visualization of cellular infiltrates (red, upper panel). Representative images of the four cohorts are depicted. Rectangle in A indicates area of higher magnification for all images (middle panel). Cross sections were also stained with DAPI to visualize cellular infiltration (lower panel). Dotted line indicates area of grey matter determined by an overlay image of the consecutive LFB stained cross section. **(B)** Area of demyelination was measured in 2 cross section/animal in all 4 groups (n = 4–6) as percentage of red infiltration areas related to general white matter area. Area was determined using the area measuring tool of the Fiji/ImageJ 1.46j software. **(C)** Number of DAPI positive cells within the white matter of 3 cross sections/animal in the 4 cohorts (n = 4). Cells were quantified using the automatic counting tool of the Fiji/ImageJ 1.46j software. EAE = immunized mice, untreated; CU ctrl. = *C*. *utilis* wild-type; CU MOG = CBCu17; CU MOG HK = CBCu17, heat killed. Depicted is the mean ± SEM. Asterisks indicate significance (* p < 0.05; *** p < 0.01 *** p < 0.001) student’s t test.

The integrity of myelin (blue) and the inflammation (red) of the CNS spinal cords of the four cohorts (day 35 p. i.) was visualized (Fig 4A, upper panel). Representative images of spinal cord revealed higher myelin integrity and a reduced number of areas with massive cellular infiltration into the white matter in mice receiving the MOG_35-55_ peptide on living or heat inactivated *C*. *utilis* compared to controls. Images of higher magnification, indicated by the rectangle in Fig 4A (middle panel), also revealed that overall scattered infiltration is reduced in MOG treated animals. Cellular infiltration was also visualized using DAPI to stain cell nuclei (Fig 4A, lower panel). Dotted line indicates white matter, determined by overlays of luxol fast blue stained and DAPI stained consecutive images. Again, the cellular infiltration was more prominent in the control groups.

Quantification of demyelinated area in percentage of white matter (Fig 4B) and cellular infiltration of white matter (Fig 4C) confirmed that control animals with EAE or after oral administration of wild type *C*. *utilis* showed significant higher cellular infiltration and more demyelinated areas compared to animals were MOG is present in the gastrointestinal tract.

### Reduced T cell response to MOG and higher regulatory T cell numbers in mice fed with MOG_35-55_ expressing *C*. *utilis*

**Fig 5:**
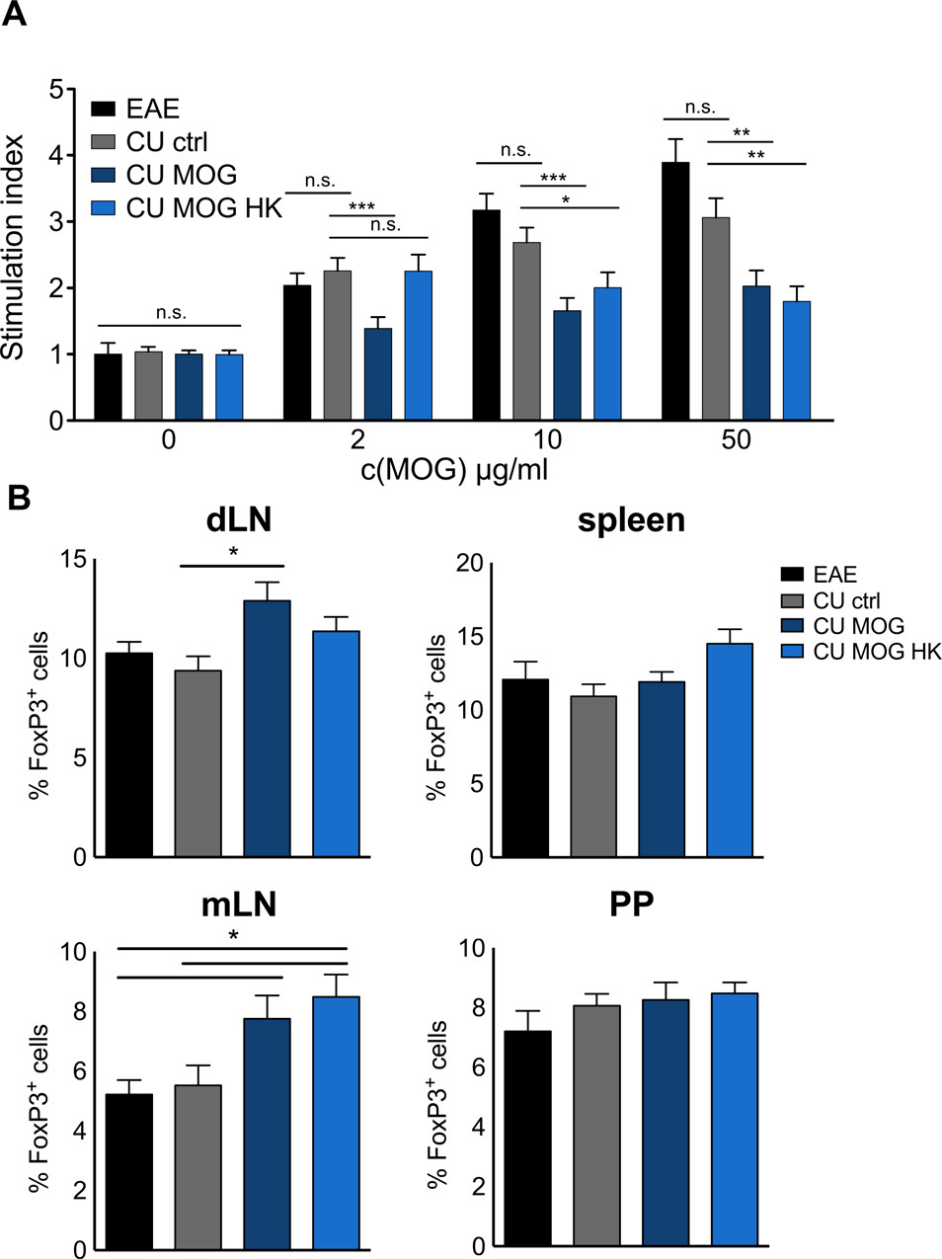
T cell proliferation is reduced in MOG_35-55_ treated animals. **(A)** T cell proliferation in response to rechallenge with MOG_35-55_ at the indicated concentrations. Spleen cell proliferation at day 15 of EAE of animals of the indicated groups was measured by [3]H-thymidine incorporation in the presence of increasing concentrations of MOG_35-55_ (0–50 μg/ml) in quadruplicates of four independent experiments. Depicted is the mean ± SEM of six mice per group. **(B)** Number of FoxP3 positive cells gated on CD4 T cells of lymphoid organs. Cells from the indicated organs were prepared at day 15 of EAE from the four groups. Intranuclear FoxP3 staining of was used to determine frequencies of regulatory T cell within the CD4 population in three independent experiments with 4 mice per group. dLN: immunization site draining lymph node; mLN: mesenteric (gut-draining) lymph node; PP: Peyer’s patches. EAE = immunized mice, untreated; CU ctrl = *C*. *utilis* wild-type; CU MOG = CBCu17. Asterisks indicate significance (* p < 0.05; *** p < 0.01 *** p < 0.001) student’s t test.

The antigen specific T cell proliferation was considerably increased in splenocytes with increasing MOG_35-55_ concentrations from mice either immunized or treated with wild type *C*. *utilis* (Fig 5A) compared to control without antigen. In contrast, mice receiving heat killed or living with MOG_35-55_ expressing *C*. *utilis* only moderately respond to autoantigenic rechallenge. The concentration of the proinflammatory cytokine interleukin (IL)17 was not significantly altered in supernatants of the T cell proliferation assay (data not shown). To determine differences in the number of regulatory T cells, lymphoid organs were stained for intranuclear FoxP3 (Fig 5B). The frequencies of regulatory T cells were not significantly altered in the spleen and the Peyer’s patches (PP) of the four groups. However, immunization site draining lymph nodes (dLN) of mice receiving MOG_35-55_ expressing living *C*. *utilis* contained significantly more FoxP3^+^ cells than mice fed with the *C*. *utilis* control strain. In the mesenteric lymph node the number of regulatory T cells was significantly higher in mice receiving *C*. *utilis* stains expressing MOG than in the two control groups (Fig 5B).

## Discussion

Safe and low-cost applications are highly desirable to induce oral tolerance in organisms. While the administration of purified antigens, e.g. MOG, MBP, OVA, to induce immunological tolerance is an expensive and time intensive method, the use of microorganisms presenting antigens on their surfaces is a promising tool for the future—not only with regard to auto immune diseases but also for oral vaccination. Yeast and bacterial strains expressing antigens were already successfully used in several studies to either induce an antigen specific immune answer [23–26] or to induce mucosal tolerance [9–12]. In these studies oral tolerance was induced by feeding mice with either a purified immunogenic epitope, by feeding soluble cell extracts of bacteria producing this epitope or by feeding unimpaired bacteria producing immunogenic epitopes intracellular [9–12]. Induction of peripheral tolerance against a specific peptide is possible not only through the GI-tract but through many different ways. Recent studies showed that injection of the immunogenic MOG_35-55_ peptide in the ocular anterior chamber of the eye induced generation of splenic CD4^+^ and CD8^+^ regulatory T cells and suppressed EAE in mice [27,28]. In another study, Farooq and Ashour [29] showed, that intravenous injection of *in vitro*-generated B-cells, Tregs and APCs specific to the MOG_35-55_ epitope induces peripheral tolerance in mice. Even the administration of engineered, tolerogenic synthetic peptides was successfully used in preventing EAE in mice [30]. Although, these are all established methods to induce peripheral tolerance, we were using a different, non invasive and cost-efficient method by which the antigen can be administered easily to animals and, in the future, to human patients.

Here, we used *C*. *utilis*, a food yeast, expressing the immunogenic MOG_35-55_ epitope on its surface for the induction of an oral tolerance. Previously, the fusion of DNA sequences encoding heterologous proteins to the secretion and GPI addition signal sequences of the *C*. *utilis GAS1* sequence had been shown to be translated into functional proteins present on the cell surface (cell wall) of *C*. *utilis* [19,20]. Cell associated MOG in CBCu8 was expected at a size of about 21 kDa but was detected at a size of about 27 kDa (Fig 2A). The increased mass may be due to attached ß1,6-glucan of cell wall-bound protein or to the presence of GPI anchor in the protein precursor in transit to the cell wall. This was also observed in surface displays with other heterologous proteins [19].

We tested whether the MOG-presenting *C*. *utilis* strain was able to reduce the severity of the autoimmune disease of the CNS in the mouse model EAE. We observed that *C*. *utilis* is not able to grow in the gut and established a feeding protocol which indicated that a minimal amount of 1x10^8^ cells is needed to pass the gut. Even a second, continuous feeding scheme for 5 days did not lead to a colonization of the gut as in histological samples only in the caecum *C*. *utilis* was found. This was described as the preferential localisation of yeasts in the gut [31]. Although the exact amount of MOG provided by this carrier was not known, the clinical course of EAE was ameliorated (Fig 3).

The significantly reduced clinical course of the groups that received the MOG_35-55_ expressing *C*. *utilis* strains is a combination of a lower incidence as well as a maximum score reduction. The presence of MOG in the gut protected the mice from EAE and additionally the mice that developed EAE had a milder disease course. Most interestingly there was no difference in feeding alive or heat-inactivated *C*. *utilis*, in contrast, the feeding of heat killed *C*. *utilis* expressing MOG seems to be more efficient. If this is due to the fact that killing *C*. *utilis* may induce a higher availability of MOG by the release of intracellular MOG has to be further elucidated. The clinical data were endorsed by Luxol staining of spinal cords (Fig 4). The protection of the myelin and the reduced cellular infiltrates are in line with the clinical score and underline the efficiency of MOG_35-55_ expressing *C*. *utilis* for protection against the autoimmune disorder of the CNS, most likely by the induction of oral tolerance. Additionally, the T cells are less responsive to autoantigenic rechallenge when mice were fed with MOG_35-55_ expressing *C*. *utilis* (Fig 5A). In the supernatants of the proliferation assay the amount of the proinflammatory cytokine IL17 was not altered in the four groups. However, we detected a significant increase in regulatory T cells in the lymph nodes, especially in the mesenteric lymph nodes draining the gut indicating the successful induction of oral tolerance by feeding of MOG expressing *C*. *utilis* (Fig 5B).

Hence, the point that *C*. *utilis* is not colonizing the gut and is excreted directly may be an advantage, because the microbiome is not affected and the administration of antibiotics is not necessary.

Oral tolerance induction is a well structured orchestration of mechanisms dampening the inflammatory response against (auto)antigens, however, many blind spots still remain. It affects the number and function of regulatory cells, amount of anti-inflammatory and proinflammatory cytokines and regulates the number of effector cells like TH1 and TH2 [32]. For some cytokines the inflammatory profile is still discussed. Granulocyte-Macrophage colony stimulating factor (GM-CSF) is besides its role as hematopoietic growth factor long considered to be a proinflammatory cytokine. When given intracerebroventricularly in healthy mice, it induces the massive infiltration of fully competent myeloid DCs in the CNS [33]. In CNS autoimmunity this cytokine produced by T cells drives myeloid cells to a proinflammatory phenotype propagating tissue damage [34]. Other papers discuss the role of GM-CSF in autoimmune tolerance, given the fact that low-dose GM-CSF is able to act on tolerogenic DCs leading to the mobilization of IL-10 secreting Tregs [35]. To what extent GM-CSF is induced by feeding mice with MOG expressing *C*. *utilis* has to be further elucidated.

Oral (mucosal) T cell tolerance is known to induce multiple mechanisms controlled by the dose of antigen and feeding regimen. While high doses of antigen preferentially induce anergy or deletion of antigen-specific immune cells via apoptosis, low dosages favour active suppression through induction of antigen-specific Tregs [36–39]. They produce high amounts of anti-inflammatory cytokines after encountering their cognate antigen and can induce a milieu also suppressing bystander cells [40]. This process could subsequently lead to inhibition of autoimmune responses not only against the responsible autoantigen but also to different antigens within cells in close proximity. In the present study, the rechallenge with autoantigens in a T cell proliferation assay was reduced indicating that the overall immune response to the autoantigen is ameliorated. To what extend the protection of myelin is induced via the induction of an anti-inflammatory milieu that also protects against other autoimmune targets has to be further elucidated. A broader myelin protection than to the specific administered autoantigen would be of special interest since autoimmune targets in MS are not completely defined.

## Conclusion

Collectively, our results indicate that MOG-presenting viable and heat-inactivated *C*. *utilis* cells are able to generate an efficient myelin protection against CNS autoimmunity by the induction of oral tolerance. An ongoing approach is to use the recombinant *C*. *utilis* strain also in models of relapsing remitting MS to explore, if its tolerogenic capacity can also been used between relapses, within the asymptomatic phase of chronic MS disease.

## Supporting Information

S1 Fig chromosomal integration and stability of pCB10 in *C*. *utilis*. **(A)** Genomic *TDH3* locus of *C*. *utilis* DSMZ2361. gDNA was restricted with *Kpn*I and a *TDH3*p probe was used to detect DNA fragments. In *C*. *utilis* wild-type an 8.6 kb band is expected, when plasmid pCB10 is integrated in one of the *TDH3* alleles, sizes of 3.9 kb and 11.3 kb are detected. **(B)** Southern analysis of *Kpn*I digested gDNA of *C*. *utilis* wild-type and three (1, 2, 3) CBCu8 transformants. Plasmid DNA sequences were detected using a *TDH3*p probe. Plasmid pCB10 (500 ng) was used as a positive control. The chromosomal 8.6 kb *TDH3*p band is indicated by a black triangle, plasmid specific 3.9 kb and 11.3 kb *TDH3*p sequences are indicated by a white and grey triangle, respectively. **(C)** Plasmid stability of CBCu8. Strain CBCu8 was incubated for 50 generations in YPD medium either with (+N) or without (-N) 10 μg/ml Nourseothricin (NST). Cells were washed, diluted and plated out on YPD agar plates. 100 cells were then spotted on agar plates supplemented with 10 μg/ml NST. After 2 d at 30°C cells were counted and plasmid stability was calculated. Mean and SEM of three independent replicates is shown. Asterisks indicate significance (* p < 0.05) in student’s t test.
(PPTX)
