# Supplementary material for: Oral Tolerance Induction in Experimental Autoimmune Encephalomyelitis with Candida utilis Expressing the Immunogenic MOG35-55 Peptide
Source: PLoS One. 2016 May 9;11(5):e0155082. doi: 10.1371/journal.pone.0155082 (PMC4861260; doi:10.1371/journal.pone.0155082)
Supplement: S1 Fig — (A) Genomic TDH3 locus of C. utilis DSMZ2361. gDNA was restricted with KpnI and a TDH3p probe was used to detect DNA fragments. In C. utilis wild-type an 8.6 kb band is expected, when plasmid pCB10 is integrated in one of the TDH3 alleles, sizes of 3.9 kb and 11.3 kb are detected. (B) Southern analysis of KpnI digested gDNA of C. utilis wild-type and three (1, 2, 3) CBCu8 transformants. Plasmid DNA sequences were detected using a TDH3p probe. Plasmid pCB10 (500 ng) was used as a positive control. The chromosomal 8.6 kb TDH3p band is indicated by a black triangle, plasmid specific 3.9 kb and 11.3 kb TDH3p sequences are indicated by a white and grey triangle, respectively. (C) Plasmid stability of CBCu8. Strain CBCu8 was incubated for 50 generations in YPD medium either with (+N) or without (-N) 10 μg/ml Nourseothricin (NST). Cells were washed, diluted and plated out on YPD agar plates. 100 cells were then spotted on agar plates supplemented with 10 μg/ml NST. After 2 d at 30°C cells were counted and plasmid stability was calculated. Mean and SEM of three independent replicates is shown. Asterisks indicate significance (* p < 0.05) in student’s t test. (PPTX) [file pone.0155082.s001.pptx]

## Slide 1
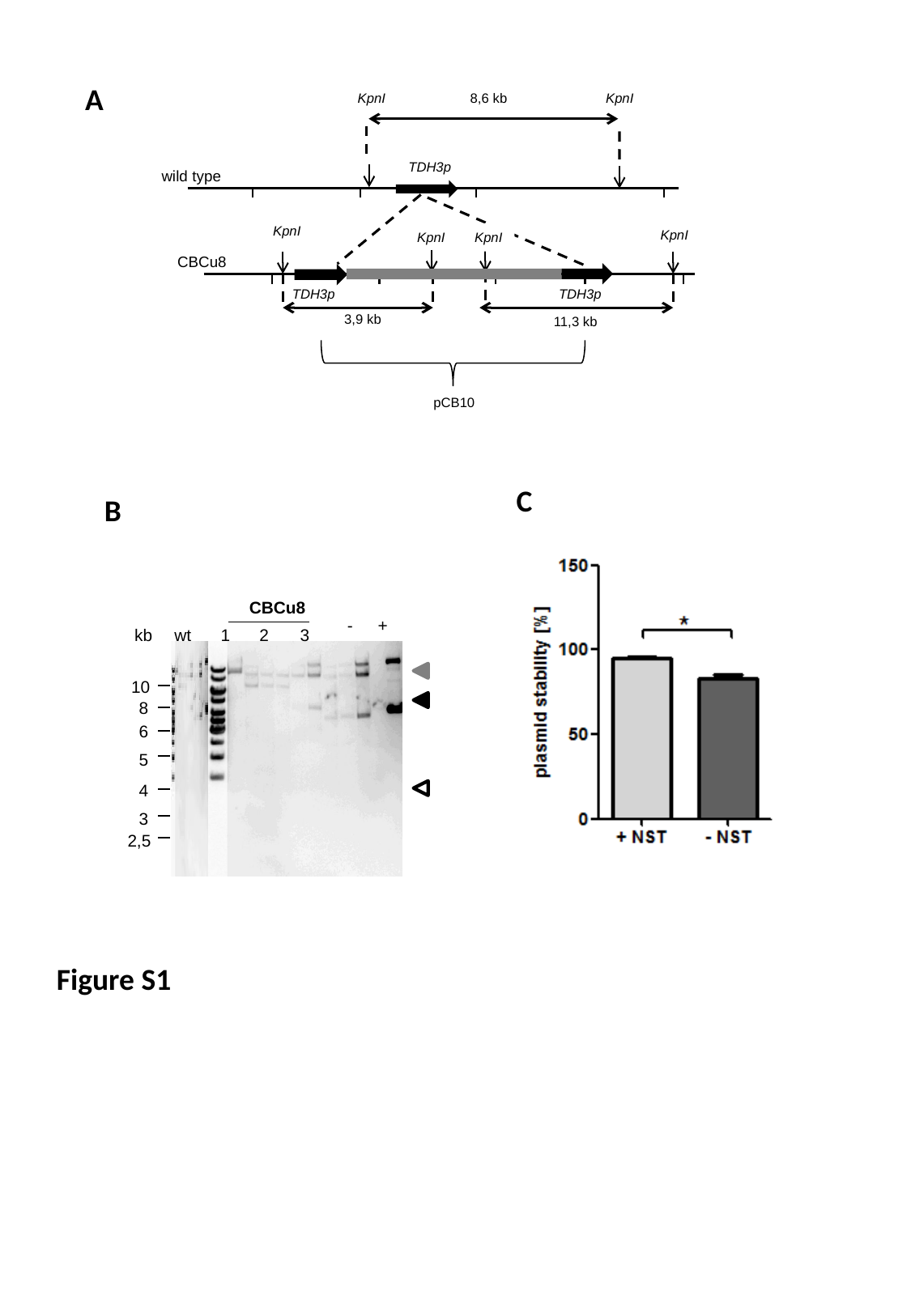

A
KpnI
KpnI
TDH3p
KpnI
KpnI
KpnI
TDH3p
TDH3p
3,9 kb
11,3 kb
pCB10
KpnI
8,6 kb
wild type
CBCu8
C
B
CBCu8
-
+
kb
wt
1
2
3
10
8
6
5
4
3
2,5
Figure S1
